# The Potential of 2-Substituted Quinolines as Antileishmanial Drug Candidates

**DOI:** 10.3390/molecules27072313

**Published:** 2022-04-02

**Authors:** Philippe M. Loiseau, Kaluvu Balaraman, Gillian Barratt, Sébastien Pomel, Rémy Durand, Frédéric Frézard, Bruno Figadère

**Affiliations:** 1 Antiparasite Chemotherapy, CNRS, BioCIS, Université Paris-Saclay, 92290 Chatenay-Malabry, France; sebastien.pomel@universite-paris-saclay.fr (S.P.); remy.durand@universite-paris-saclay.fr (R.D.); 2 Chemistry Department, Georgetown University, 37th and O Streets, Washington, DC 20057, USA; bala04chem05@gmail.com; 3 Institute Galien Paris-Saclay, CNRS, Université Paris-Saclay, 92290 Chatenay-Malabry, France; gillian.barratt@universite-paris-saclay.fr; 4 Department of Physiology and Biophysics-ICB, Universidade Federal de Minas Gerais, Belo Horizonte 31270-901, Brazil; frezardf@gmail.com; 5 Chimie des Substances Naturelles, CNRS, BioCIS, Université Paris-Saclay, 92290 Chatenay-Malabry, France; bruno.figadere@universite-paris-saclay.fr

**Keywords:** antiparasitic drugs, 2-substituted quinolines, leishmaniasis, antileishmanial agents, mechanism of action, drug targeting

## Abstract

There is a need for new, cost-effective drugs to treat leishmaniasis. A strategy based on traditional medicine practiced in Bolivia led to the discovery of the 2-substituted quinoline series as a source of molecules with antileishmanial activity and low toxicity. This review documents the development of the series from the first isolated natural compounds through several hundred synthetized molecules to an optimized compound exhibiting an in vitro IC_50_ value of 0.2 µM against *Leishmania donovani*, and a selectivity index value of 187, together with in vivo activity on the *L. donovani*/hamster model. Attempts to establish structure–activity relationships are described, as well as studies that have attempted to determine the mechanism of action. For the latter, it appears that molecules of this series act on multiple targets, possibly including the immune system, which could explain the observed lack of drug resistance after in vitro drug pressure. We also show how nanotechnology strategies could valorize these drugs through adapted formulations and how a mechanistic targeting approach could generate new compounds with increased activity.

## 1. Introduction

Leishmaniases are a family of neglected tropical and sub-tropical diseases caused by flagellated protozoan parasites of the *Leishmania* genus that are transmitted by the bite of infected phlebotomine sandflies [1]. About twenty *Leishmania* species can infect humans, resulting in three main types of leishmaniasis. The visceral leishmaniasis (VL) form known as kala-azar is lethal in the absence of treatment. Its symptoms are anarchic fever accompanied by a significant pallor due to anemia, weight loss and, occasionally, abdominal pain. The spleen (significant splenomegaly), liver (moderate hepatomegaly) and lymph nodes (lymphadenopathy) all increase in volume [2]. The most common form is cutaneous leishmaniasis (CL), presenting with cutaneous lesions, while the mucocutaneous leishmaniasis (MCL) form provokes deep lesions that are prone to bacterial superinfection. Leishmaniases mainly affect poor people in Africa, Asia and Latin America, and 98 countries were endemic for leishmaniasis in 2021 [1]. The majority of VL cases occur in eight countries: Brazil, Eritrea, Ethiopia, India, Kenya, Somalia, South Sudan and Sudan; CL cases are predominant in Afghanistan, Algeria, Brazil, Colombia, Iraq, Pakistan and Syria; and MCL cases are present in South America. Approximately 1 billion people are thought to live in areas endemic for leishmaniasis, and more than 10,000 new cases of VL occur every year, according to the WHO [3]. Some relapses of visceral leishmaniasis can lead to post-kala-azar dermal leishmaniasis, while disseminated leishmaniasis is a severe form of American tegumentary leishmaniasis [2]. Apart from some strictly anthroponotic species, such as *L. donovani* and *L. tropica*, all *Leishmania* species are zoonotic. Thus, *L. infantum* infects dogs in the Mediterranean basin and South America. The distribution of the insect vector depends on climate changes and environmental criteria such as urbanization, whereas the number of leishmaniasis cases is related to malnutrition, immunosuppression and population movements.

**Figure 1:**
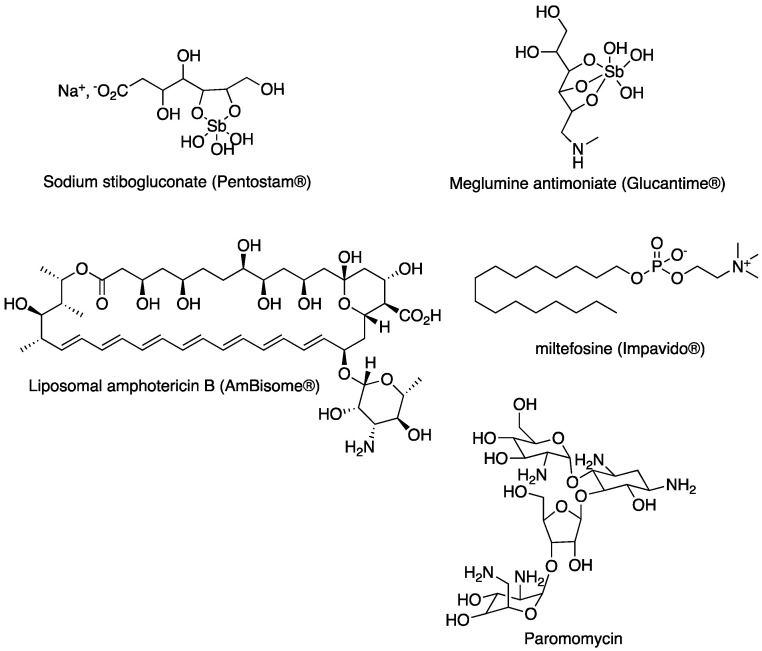
Antileishmanial drugs most currently used in clinics.

Despite considerable progress in the understanding of the molecular biology of *Leishmania* sp., antileishmanial chemotherapy remains limited to a few chemical series. Thus, for the last 70 years, pentavalent antimony compounds such as sodium stibogluconate (Pentostam^®^, GSK) and meglumine antimoniate (Glucantime^®^, Sanofi) have been the first-line treatment for leishmaniases [4]. Parenteral alternatives to antimonials such as liposomal amphotericin B (AmBisome^®^, Gilead) have fewer side effects, while miltefosine (Impavido^®^, Zentaris) was the first orally active antileishmanial drug to be developed. Paromomycin can also be used, but resistance is developing rapidly, meaning that it is used only in drug combinations [4]. These currently used drugs are presented in Figure 1.

In general, antiparasitic drugs lack specificity and therefore evoke side effects. Therefore, there is a crucial need for new chemical series that can act selectively against the parasites, ideally without the risk of drug resistance. However, the development of new drugs is increasingly difficult due to increasingly rigorous pharmacological requirements. Thus, scientific studies regularly highlight new risk factors that need to be taken into account in a risk–benefit analysis. In addition, the estimated research and development investment needed to bring a new chemical entity to market is around USD 985 million, jeopardizing the return on investment in the case of neglected diseases that affect poor countries [5].

Among the various chemical scaffolds investigated worldwide, benzoxaborole, nitroimidazole and aminopyrazole have emerged as promising series which have provided some drug candidates with the help of the Drug for Neglected Diseases initiative [6]. Thus, DNDI-6148, a novel benzoxaborole, and DNDI-0690, a nitroimidazole derivative, are now preclinical candidates for the treatment of visceral leishmaniasis thanks to their promising profile [7,8]. In parallel, two proteasome inhibitors, compound GSK 3186899, a pyrazolopyrimidine derivative [6], and compound LXE408, [9] a triazolopyrimidine derivative, are now in preclinical development.

Quinolines are ubiquitous heterocyclic nitrogen used as components of dyes, or solvents for resins, and in the production of various chemical products, including pesticides [10]. In addition, the quinoline scaffold, first discovered in natural substances, is present in a wide variety of biologically active compounds of both synthetic and natural origin. After presenting some promising results obtained with the 4-aminoquinoline [11] and 8-aminoquinoline [12] series, this review focuses on investigations performed on the 2-substituted quinoline series and its ability to provide drug candidates for the treatment of leishmaniases.

## 2. The Place of Quinolines as Drug Candidates in the Treatment of Leishmaniases

Historically, quinolines have been among the most effective antimalarial drugs [13]. Quinine, extracted from quinquina bark, was the first isolated natural compound to be used for the treatment of malaria. Many quinine derivatives were subsequently synthesized, leading to chloroquine, the use of which became prominent in the early 1950s, through the World Health Organization program to fight malaria. Thus, chloroquine rapidly became the first-line treatment for this disease, saving millions of lives in endemic areas, until drug resistance began to limit its usefulness [14]. As well as chloroquine, the quinoline series contains a number of other compounds that are very efficient against malaria, including mefloquine, amodiaquine and primaquine [13]. 

**Figure 2:**
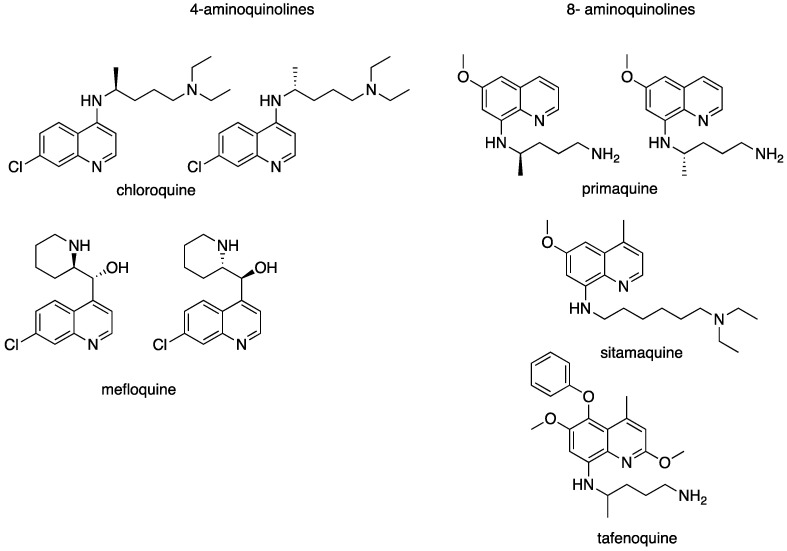
Aminoquinolines with antileishmanial activity.

More recently, attention has been devoted to the possibility of repurposing antimalarial drugs for the treatment of leishmaniases [15]. The chemical formulae of some quinolines that are active against leishmaniasis are shown in Figure 2.

Among the 4-substituted quinolines, chloroquine and mefloquine are active in vitro against *L. amazonensis* intracellular amastigotes [15]. In a clinical trial, chloroquine appeared to be as effective as tetracycline for the treatment of cutaneous leishmaniasis [16]. Combination therapy with agents possessing different mechanisms of action has long been investigated, with the aim of obtaining synergistic effects and delaying the emergence of drug resistance. In this respect, chloroquine combined with diminazene merited further development [17]; however, the combination with paromomycin did not yield encouraging results [18]. Several chloroquine derivatives have been evaluated on *Leishmania* models in vitro and in vivo [19]. However, the major source of drug candidates was the 8-aminoquinoline series. A first attempt was to reconsider primaquine by using drug carriers such as nanoparticles to concentrate the drug within the liver where the leishmania parasites are located in order to diminish its problematic toxicity. Positive results with efficient and nontoxic formulations were obtained in vitro and on the *L. donovani*/BALB/c mouse model [20,21]. However, the use of nanotechnology would add to the final cost of the drug, which could restrict its use in developing countries. Therefore, no further investigations were carried out in this direction.

Sitamaquine is an 8-aminoquinoline that has been considered as a potential drug candidate because of its aqueous solubility, antileishmanial activity by the oral route and ADME data compatible with drug development [22,23,24]. One advantage of sitamaquine is its short elimination half-life that could prevent the rapid emergence of resistance [23]. Studies of its mechanism of action have revealed an affinity for lipid membranes [25]. It accumulates rapidly within the parasite by diffusion along an electro-chemical gradient and is concentrated in the cytosol by an energy- and sterol-independent process. The binding of sitamaquine to membranes was found to be transitory, and an energy-dependent efflux was observed. This suggests the intervention of a transporter, but this has not yet been characterized [26]. Sitamaquine provokes oxidative stress in *Leishmania donovani* promastigotes by targeting mitochondrial succinate dehydrogenase [27]. In addition, susceptibility to sitamaquine does not seem to be mediated by drug accumulation in acidocalcisomes [28]. Since no exploitable activity was observed in experimental cutaneous leishmaniasis, this compound was further developed for VL treatment only [29]. It reached phase 2 clinical trials in humans, but adverse effects such methemoglobinemia and nephrotoxicity stopped further development [30,31,32]. Furthermore, a sitamaquine-resistant clone of *L. donovani* was easily selected by in vitro pressure in the laboratory; despite the absence of cross-resistance with other antileishmanial drugs, the risk of emergence of sitamaquine resistance is not negligeable, limiting further pharmaceutical development [33,34]. 

Another 8-aminoquinoline in clinical development for the treatment of malaria is tafenoquine [35]. This drug also exhibits antileishmanial activity in vitro against several *Leishmania* species, and in vivo in the *L. donovani*/BALB/c mice model, with 50% effective dose (ED_50_) values of 1.2 to 3.5 mg/kg for 5 days [36]. Tafenoquine targets respiratory complex III and provokes apoptosis [37]. Its uptake by *Leishmania* follows a sterol-dependent diffusion process [38], and it causes increased glycolytic ATP synthesis [39].

As well as these 4- and 8-aminoquinoline series, many other derivatives have also been reported to exhibit antileishmanial activity: for example, 6-methoxy-4-methyl*-n-*[6-(substituted-1-piperazinyl)hexyl]-8-quinolinamines [40]. In this context, the 2-substituted quinoline series emerged from an ethnopharmacological study in the 1990s and became a subject of research as a source of new antileishmanial drugs.

## 3. The Potential of 2-Substituted Quinolines as Antileishmanial Agents

### 3.1. From the Plant to Experimental Models of Leishmaniasis

The group of Alain Fournet, from the IRD (Institut de Recherche sur le Développement, France), observed that Bolivian traditional practitioners used extracts from the bark of *Galipea longiflora*, a tree of the Rutaceae family, to treat cutaneous lesions of leishmaniasis both as ointments and by infusing the stem bark to make decoctions for drinking. The extraction of compounds from *Galipea longiflora* bark led to the identification of about 10 original structures with a quinoline scaffold substituted at the 2-position [41,42]. Of these compounds, 2*-n-*propylquinoline, 2-propenylquinoline and 2-*trans*-epoxypropylquinoline exhibited moderate in vitro activity on promastigotes of several *Leishmania* species, with IC_50_ values in the range of 100 to 250 µM; values that are high due to their poor aqueous solubility [43]. Among other compounds identified in the leaves, 2-phenylquinoline and 2-pentylquinoline were also moderately active in vitro, while another active compound, 4-methoxy-2-phenylquinoline, was found in both the bark and roots. These compounds were next evaluated in vivo on the *L. amazonensis* and *L. venezuelensis*/BALB/c mouse models by subcutaneous or intralesional routes at 100 mg/kg/day for 14 consecutive days [44]. In these models, animals treated with 2*-n-*propylquinoline or 2-*trans*-epoxypropylquinoline according to this regime showed the same reduction in lesion size as those treated with the reference drug, Glucantime^®^, administered as subcutaneous injections at 56 mg Sb/kg/day. Despite the high dose of the tested compounds, no sign of toxicity was detected. In addition, treatment with the same dose of 2-propenyl quinoline, either by the oral route or intralesionally, for four to six weeks after infection led to a 95% reduction in the parasite load [42]. This activity was confirmed in different in vivo protocols using lower doses in *L. amazonensis* and *L. venezuelensis* BALB/C mouse models. Treatment with the quinolines was administered for 4 to 6 weeks post-infection either by the oral route at 50 mg/kg twice daily for 15 days or by five intralesional injections at intervals of 4 days with a quinoline at 50 mg/kg of body weight. The reference drug, *N*-methylglucamine antimoniate (Glucantime^®^), was administered by subcutaneous or intralesional injection (regimes of 14, 28 or 56 mg of pentavalent antimony [Sb^v^] per kg of body weight daily). In this study, 2-*trans*-epoxypropyl quinoline was the most active compound: reducing the lesion weight and parasite burden by 70–95% [44]. 

As far as VL is concerned, the antileishmanial activity of four 2-substituted quinoline alkaloids, including 2*-n-*propylquinoline and 2-*trans*-epoxypropyl quinoline, was studied in the *L. donovani* BALB/c mouse model [45]. Subcutaneous treatment with 2-*trans*-epoxypropyl quinoline for 10 days at 100 mg/kg/day resulted in an 87% parasite reduction in the liver, whereas oral administration of 100 mg/kg of 2*-n-*propylquinoline once daily for 5 or 10 days reduced parasite burdens in the liver by 88% and almost 100%, respectively. This study was the first to demonstrate the activity of 2-substituted quinoline alkaloids in the experimental treatment of visceral leishmaniasis. Another study in the same model showed that oral administration of 2*-n-*propyl quinoline and 2-*trans*-epoxypropyl quinoline at 50 mg/kg/day for five consecutive days led to reductions in the parasitic load of 87% and 70%, respectively. Furthermore, a ten-day treatment with 2*-n-*propyl quinoline resulted in a reduction in the parasite burden of 99% [45]. 

The final selection of the potential drug candidate used chemical stability and acute oral toxicity as the main discriminating criteria. On this basis, 2-*trans*-epoxypropyl quinoline and (2-(2-methoxyethenyl)quinoline) were excluded from further development. Finally, 2*-n-*propylquinoline was chosen among the natural 2-substituted quinolines isolated from *Galipea longiflora* for further studies as an antileishmanial agent [46]. It was the most stable compound under a variety of conditions and only caused reversible toxicity after treatment by the oral route at the single dose of 1000 mg/kg, while no sign of toxicity was detected at 100 mg/kg. It is noteworthy that 2-substituted quinolines were active on a *Leishmania donovani* line that was resistant to sitamaquine, an 8-aminoquinoline. This suggests that 2-substituted quinolines and 8-aminoquinoline have at least one different target in *L. donovani* [46]. Moreover, six natural 2-substituted quinolines were also active in vivo in the *Plasmodium vinckei petteri*/BALB/c mouse model after a single oral treatment at 50 mg/kg [47].

### 3.2. From Natural Compounds to Synthetic Derivatives and Their Biological Evaluation

All the compounds cited above were synthesized in the laboratory in order to obtain the quantities necessary to perform in vivo evaluations in different *Leishmania* species as well as ADME studies [48]. For example, 2*-n-*propylquinoline could be prepared in good yields by two different approaches (Figure 2). Starting from quinoline *N*-oxide, the addition at room temperature of chloroformyl isobutanolate followed by the addition at low temperature of *n*-propylmagnesium bromide led to the desired compound with a 67% yield [49]. However, when 2-chloroquinoline was treated directly with *n-*propylmagnesium bromide in the presence of a catalytic amount of Fe(acac)_3_, 2*-n-*propylquinoline was obtained with a 95% yield [50]. Several other compounds were subsequently obtained, using either the iron-catalyzed approach [51,52,53] or N-oxide quinoline transformation [52,53,54,55,56]. Around 150 compounds were prepared and screened in both in vitro and in vivo models of leishmaniases [49,50,51,52,53,54,55].

**Figure 3:**
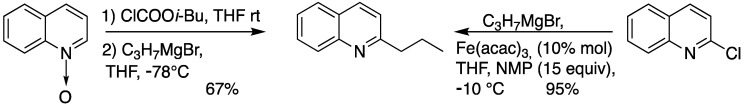
Chemical synthesis of 2*-n-*propylquinoline.

This synthesis is quite simple, requiring only a few steps and returning good yields. As an example, the synthesis of 2*-n-*propylquinoline is presented in Figure 3.

Starting from this lead compound, pharmacomodulation was carried out, resulting in a library of more than 150 compounds designed to establish structure–activity relationships and thereby optimize the series [49,50,51,52,53,54,55].

This process resulted in compounds that are more than 10 times more active in vitro than 2*-n-*propylquinoline [55]. IC_50_ values in the range of 2–4 µM were obtained on intramacrophage amastigotes of *L. donovani* and *L. infantum* in vitro*,* and in vivo, the parasite burden was reduced by about 60–70% after an oral treatment at 12.5 mg/kg for 10 consecutive days in the *L. donovani*/BALB/c mouse model [55]. These compounds were also active in vivo against *L. infantum* and *L. amazonensis*. The most promising were 2-(2-hydroxyprop-2-enyl)quinoline, and (*E*)-3-quinolin-2-yl-acrylonitrile [55,56,57] (Table 1).

Among many other compounds synthesized, some were found to be active in vitro but not in vivo. This is the case for a series of 18 styrylquinolines for which the 7-aroylstyrylquinoline scaffold appeared to be the most promising, with the most active compound, exhibiting a 50% inhibitory concentration of 1.2 μM and a selectivity index value of 121.5 [62]. This compound was 10-fold and 8-fold more active than miltefosine and sitamaquine, the reference compounds, with 607-fold and 60-fold higher selectivity indexes, respectively. However, these encouraging results in vitro were not confirmed in vivo [62]. Another study on styrylquinolines reported the in vitro activity of the original styrylquinolines on *L. panamensis* [63]. In parallel, other series have been prepared with the aim of optimizing 2-substituted quinolines with similar biological properties [64,65,66,67]. Moreover, quinoline-2-one derivatives exhibited in vitro antileishmanial activity in the range from 1 to 15 µM [68,69,70,71].

### 3.3. Formulations of the Natural 2-n-Propyl Quinoline

Three formulations were prepared for particular in vivo applications. The first was developed to improve the aqueous solubility of the compound for oral administration [59]; the second was designed to concentrate the compound within the liver as a liposomal formulation administered by the intravenous route [72]; and the third attempted to enhance drug solubility for intravenous administration followed by a wide biodistribution by inclusion in a cyclodextrin [73].

#### 3.3.1. Preparation, Characterization and Biological Activity of a 2*-n-*Propylquinoline Salt to Improve Aqueous Solubility

Since 2*-n-*propyl quinoline is an oil, a camphor sulfonic salt designed to facilitate in vivo administration was prepared and characterized (Table 1) [59]. 

This new salt formulation did not alter the intrinsic activity, which remained similar to that of the reference oral drug, miltefosine, in the *Leishmania donovani*/BALB/c mouse model after treatment by the oral route at 10 mg/kg/day for ten consecutive days (Table 1). The salt formulation reduced the parasite burden by 76% compared with 89% for miltefosine, demonstrating the suitable druggability of 2*-n-*propylquinoline for further studies [59]. Although this 2*-n-*propyl quinoline camphor sulfonic acid salt formulation improved the conditions of oral administration, its solubility was still not sufficient for use by the intravenous route.

#### 3.3.2. Preparation, Characterization and Biological Activity of a Liposomal Formulation of 2*-n-*Propylquinoline for the Treatment of Visceral Leishmaniasis by the Intravenous Route

A liposomal formulation of the hydrophobic 2*-n-*propylquinoline was prepared to permit intravenous administration and concentrate the drug within the liver, which harbors a large proportion of the parasites during VL [72]. This formulation, denoted 2*-n-*PQ-Lip, had a particle diameter of about 160 nm and an encapsulation yield of 53% of added drug, so the final 2*-n-*propylquinoline content of the liposomes used in the biological experiments was 5% in molar proportions. The liposomal formulation exhibited activity in vitro, with IC_50_ values in the range of 3–6 µM against *L. donovani* intramacrophagic amastigotes (Table 2). 

**Figure 4:**
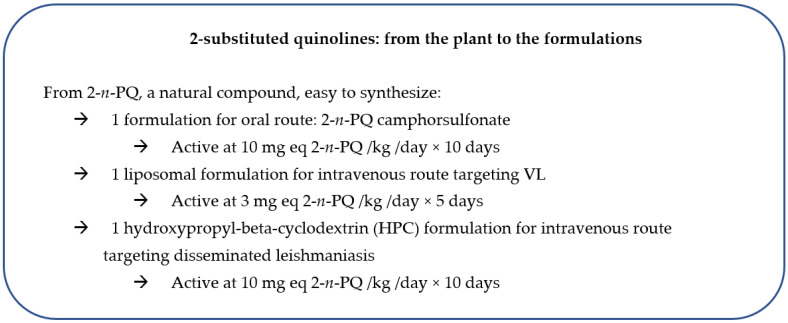
Formulations of 2*-n-*propylquinoline that merit further investigation.

Intravenous 2*-n-*PQ-Lip was active in the *L. donovani* mouse model at 3 mg equivalent 2*-n-*propylquinoline/kg/day × 5 days, a dose level that could be achieved in clinical settings. In addition, a liposomal formulation combining 2*-n-*propylquinoline at 0.75 mg/kg and amphotericin B at 6 µg/kg/day for 5 days showed a significant synergistic effect in vivo (Table 2) [72]. These results indicate the potential of 2*-n-*PQ-Lip as a promising formulation for further investigation in various leishmaniasis models (Figure 4).

#### 3.3.3. Preparation, Characterization and Biological Activity of a Formulation of 2*-n-*Propylquinoline with Hydroxypropyl Beta-Cyclodextrin for the Treatment of Different Manifestations of Leishmaniasis

Since 2*-n-*propylquinoline had good antileishmanial activity in vivo after administration by the oral route in various animal models, there was an interest to develop an intravenous formulation of 2*-n-*propylquinoline for use in disseminated leishmaniasis. However, the lipophilicity of this compound necessitates a suitable formulation for the intravenous route. With this in mind, a formulation of 2*-n-*propylquinoline with hydroxypropyl beta-cyclodextrin (2*-n-*PQ-HPC) was prepared, characterized and evaluated on *Leishmania donovani* in vitro and in vivo [73]. This formulation enhanced the in vitro activity of the compound, with an IC_50_ value of 6 µM on intramacrophagic amastigotes, and was active both on wild-type and drug-resistant parasites. An interesting point was that 2*-n-*PQ-HPC did not generate drug resistance after in vitro drug pressure. 2*-n-*PQ-HPC was also active on the *L. donovani*/BALB/c mouse model with an intravenous treatment regime of 10 mg/kg/day on 10 consecutive days, without toxicity. A pharmacokinetic study in rats after intravenous administration of the formulation at 10 mg/kg showed that the plasma concentrations of 2*-n-*propylquinoline rapidly declined bi-exponentially with a half-life of 58.7 min, and that the apparent volume of distribution was high, indicating that 2*-n-*propylquinoline was well distributed throughout the tissues, favoring parasite elimination in disseminated leishmaniasis [73]. This formulation merits further investigation on other *Leishmania* models, such as *L. infantum* in the dog for a potential veterinary development (Figure 4).

### 3.4. Entering the DNDi Pipeline to Obtain Second-Generation 2-Substituted Quinolines

**Figure 5:**
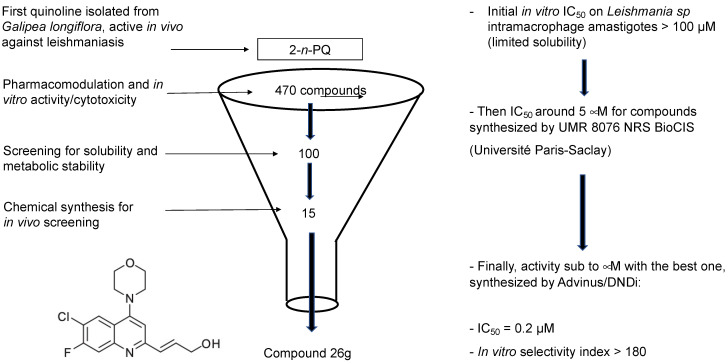
Workflow for selecting the most promising compound.

Taking into account the published data concerning the activity of 2-substituted quinolines, DNDi accepted a project aimed at optimizing the chemical series of 2-substituted quinolines in partnership with Advinus Therapeutics, Bangalore, India. Thus, a library of more than three hundred 2-substituted quinoline compounds was synthesized to identify a potential drug candidate to treat VL [61]. These compounds were evaluated for their in vitro and in vivo biological activity against *Leishmania donovani* at the CDRI, Lucknow, India, according to the workflow presented in Figure 5. The metabolic stability of these compounds with improved metabolic stability was also generated by the introduction of halogen substituents. As a result, compound 26 g (3-(6-chloro-7-fluoro-4-morpholino) quinoline prop-2-en-1-ol) was found to be the most active, with an IC_50_ value of 0.2 µM and a selectivity index of more than 180 (Table 1) [61]. The hydrochloride salt of compound 26 g showed an 84% reduction in the parasite burden after oral treatment at 50 mg/kg twice daily for 5 days in the *L. donovani* hamster model. The efficacy correlated well with the pharmacokinetic data that indicated a wide distribution of the compound. In vitro ADME characterization of the lead compound 26 g was undertaken, and some structural derivatives were synthesized and evaluated for their antileishmanial activity [74]. Compound 26 g appeared to permeate very well in the intestinal PAMPA model and was moderately bound to mouse and human plasma proteins (85–95% bound), and its blood-to-plasma concentration ratio was less than one, but it was instable in blood [74]. Compound 26 g was not a substrate of CYP450 forms CYP2C9, 2C19, 2D6 and 3A4. It showed inhibition of CYP1A2, with an IC_50_ value of 0.50 µM. Some derivatives of compound 26 g were synthesized and tested for their in vitro antileishmanial activity against *Leishmania donovani*. Since these compounds exhibited similar activity to compound 26 g, this original compound remains the drug candidate to be investigated further.

### 3.5. Structure–Activity Relationships

#### 3.5.1. Natural Compounds and Synthetic Compounds of the First Generation

When considering the 2-substituted quinolines of the first generation, a collection of about 150 compounds, there were no clear-cut structure–activity relationships emerging from the in vitro results. However, two structural parameters seem to predict the best combination of in vitro and in vivo activity: the carbon-2 substitution being an alkyl chain of three carbon atoms, with one unsaturation at the alpha or beta position [41,42,43,44,45,46,48,49,50,51,52,53,54,55,56,57]. 

#### 3.5.2. DNDi Series

**Figure 6:**
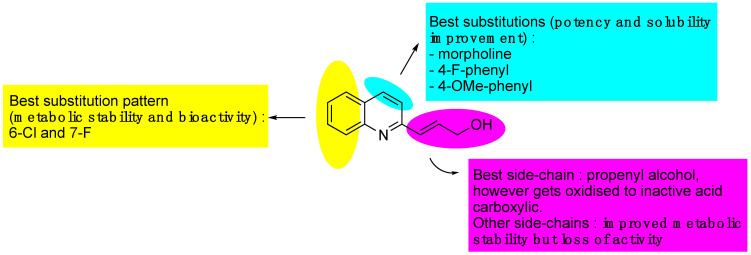
Structure–activity relationships of the 2-substituted quinolines.

Structure–activity studies were carried out with two objectives: to increase the antileishmanial activity and to improve the metabolic stability by introducing halogens, amines and aromatic rings at different positions on the quinoline ring. The structure–activity relationships can be summarized as follows: The best side chain on the carbon-2 position is a propenyl-alcohol, but this is oxidized to an inactive carboxylic acid. The best substituents on the carbon-4 position were found to be the following groups: morpholino, 4-F-phenyl and 4-OMe phenyl, providing better activity and solubility. On the carbon-6 and carbon-7, the best substitutions were 6-Cl and 7-F, improving metabolic stability rather than increasing activity [61,74] (Figure 6). Finally, all these modifications led to compound 26g: 3-(6-chloro-7-fluoro-4-morpholino) quinoline prop-2-en-1-ol, as the most potent derivative, with an IC_50_ value of 0.22 µM and a selectivity index value of 187 [61] (Table 1). This compound exhibited improved metabolic stability in human and mouse liver microsomes but not in hamster liver microsomes [61,74]. As stated above, compound 26 g reduced the parasite burden by 84% after an oral treatment at 50 mg/kg/day × 5 twice daily on the *L. donovani*/hamster model, whereas miltefosine, the reference drug, reduced the parasite burden by 96% after an oral treatment at 30 mg/kg × 5 once daily [61].

### 3.6. Mechanism of Action

The mechanism of action of small molecules such as quinolines can be determined in a number of ways. The first approach is to understand the biodistribution of 2-substituted quinolines, which requires an analytical method to quantify them. For this, a SPE/HPLC/DAD method was developed for the in vivo monitoring of several antileishmanial 2-substituted quinolines [75]. Two linear gradients were used to ensure the resolution of metabolites. The recovery of quinolines from rat plasma was in the range of 80 to 88%. From a drug development perspective, the apparent pK(a), lipophilicity and solubility were determined, as well as the extent of binding to albumin and other plasma proteins [75]. Using this method, liver microsome and hepatocyte-mediated biotransformation of some 2-substituted quinolines could be studied [76], as well as the different isoforms of rat cytochrome P450 responsible for the biotransformation of 2*-n-*propyl quinoline. Incubation of 2*-n-*propylquinoline with microsomes led mainly to hydroxylation of the side chain, involving many cytochromes: predominantly CYP2B1, CYP2A6 and CYP1A1 (at more than 80%). In contrast, minor metabolites hydroxylated on the quinoline ring involved fewer cytochromes [76]. The hydroxylated products of 2*-n-*propyl quinoline were conjugated with glucuronic acid in rat hepatocyte systems. Compounds containing a propenyl chain functionalized at the gamma position by either a nitrile or an alcohol (the latter compound being 2-(2-hydroxyprop-2-enyl)quinoline) mainly reacted with glutathione and underwent no further metabolism. However, since this reaction is reversible, the compound 2-(2-hydroxyprop-2-enyl)quinoline could be released from glutathione and underwent alternative reaction pathways [75,76]. Therefore, this analytical method revealed that the nature of the substitution on the carbon-2 position determines the metabolic routes that the compound follows [75]. Moreover, some quinolines substituted on their carbon-2 could not be detected in plasma during pharmacokinetic studies, suggesting their possible sequestration by blood components. 2-(2-Hydroxyprop-2-enyl)quinoline showed a strong affinity for red blood cells (RBCs), whereas 2*-n-*propylquinoline did not bind [58]. This binding was a saturable, temperature-dependent process and was positively correlated with the in vitro antileishmanial activity of the quinolines, with those that bound most to RBCs being the most active. A rapid and spontaneous reaction with thiol groups was demonstrated for unsaturated quinolines such as 2-(2-hydroxyprop-2-enyl)quinoline, suggesting a mechanism of binding to proteins [58]. This reactivity with RBCs could play a role in targeting compounds to the parasite, since senescent RBCs are destroyed in the spleen where parasites also are located. These results illustrate that quinoline analogues with similar antileishmanial activity in vivo can behave differently in the blood compartment. 

A series of 2-substituted aryl quinolines was synthesized and evaluated for activity against *L. braziliensis* [77]. One of them, 6-ethyl-2-phenylquinoline, was active in vitro without toxicity for macrophages. The mechanism of action described for this compound involves an alteration of parasite bioenergetics, through a disruption of the mitochondrial electrochemical potential, an alkalinization of acidocalcisomes and the inhibition of ergosterol biosynthesis in promastigote forms [77]. It is not surprising that, as small molecules, the 2-substituted quinoline series may have plurifactorial mechanisms of action. 

Some 2-substituted indolyl quinolines have been described as inhibiting the relaxation and decatenation reactions catalyzed by type I and type II DNA topoisomerases of *L. donovani* [78]. In this study, three compounds acted as inhibitors of two types of topoisomerase in *Leishmania*, with the parasitic enzymes being more susceptible to these compounds than other eukaryotic topoisomerases [78]. Unfortunately, there are no published data about the in vitro antileishmanial activity of these compounds. Since topoisomerases have been identified as interesting biological targets in *Leishmania*, it would be interesting to determine structure–activity relationships to select more specific compounds, as no other 2-substituted quinolines have yet been studied on these targets [79].

A study reported the synthesis and antileishmanial evaluation of hybrid tetrahydroquinoline and 2-substituted quinoline derivatives with phosphorated groups, on intramacrophagic amastigotes of *L. infantum* [80]. Some compounds in this series displayed an activity and a selectivity index similar to those of the standard drug amphotericin B (SI between 43 and 57). One of them showed a high degree of inhibition towards *Leishmania* topoisomerase IB. A theoretical study of their stereoelectronic properties, of the application of docking-based virtual screening methods and of the molecular electrostatic potential with predictive druggability analyses was also described [80].

Although an unpublished metabolomic analysis has been performed on two 2-substituted quinolines (Pomel S., personal communication), it did not reveal a clear over/under-expression of metabolites, suggesting a multitarget mechanism of action. A complementary pharmacoproteomic approach would yield conclusive information. In parallel, a study has reported on the relationship between the antileishmanial activity of quinolinic alkaloids from *Galipea longiflora* Krause, known as Evanta, and their effect on the immune system [81]. Thus, pretreatment of spleen cells in vitro with an alkaloid extract of Evanta (AEE) was found to interfere with proliferation and interferon-γ (IFN-γ) production in lymphocytes polyclonally activated with either concanavalin A or anti-CD3. In addition, in vitro and in vivo treatment reduced recall lymphocyte responses, as measured by IFN-γ production (55% and 63% reduction compared to untreated cells, respectively), and the production of IL-12 and TNF was inhibited. In contrast, meglumine antimoniate (SbV) did not provoke these effects. The footpad thickness and the parasite load were efficiently controlled after treatment with AEE in the *L. braziliensis* mouse model. A combination treatment of AEE and meglumine antimoniate returned better results compared with AEE or SbV alone [81]. These results suggest that it would be interesting to test the effects of pure 2-substituted quinolines from this *Galipea long*iflora extract in the immune system.

### 3.7. Drug Resistance

**Figure 7:**
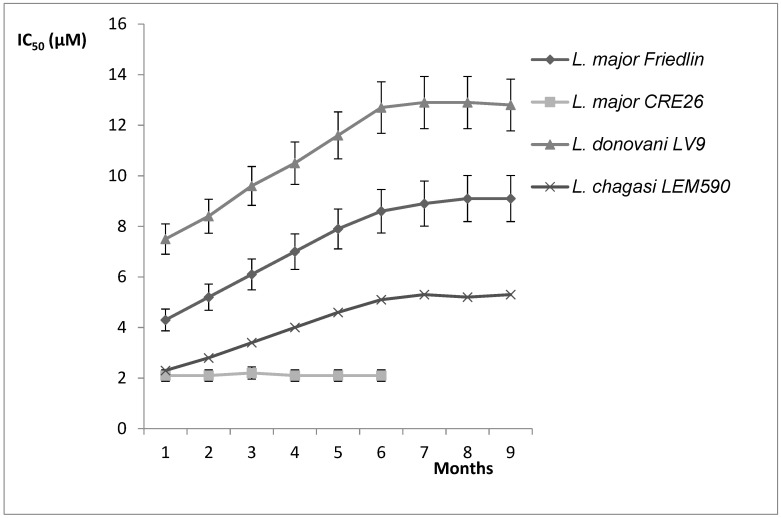
Kinetics of *Leishmania sp*. susceptibility to 2*-n-*propyl quinoline hydroxypropyl beta-cyclodextin under in vitro stepwise drug pressure [73].

In order to appreciate the risk of drug resistance, in vitro drug pressure with a hydroxypropyl beta-cyclodextrin formulation of the natural compound 2*-n-*propylquinoline was applied to promastigote cultures of *L. donovani, L. chagasi* and two strains of *L. major* [73]. This drug pressure did not lead to an increase in the IC_50_ values of the 2*-n-*propylquinoline formulation to more than twice those of the wild-type parent strains (Figure 7). With the exception of *L. major* CRE26, for which no difference in IC_50_ was observed, the drug susceptibility slowly decreased and reached a plateau after 7 months of drug pressure [73]. However, the difference in IC_50_ values could not be considered as drug resistance because the resistance index, as the ratio of IC_50_ after drug pressure/IC_50_ before drug pressure, was less than 4. This absence of drug resistance is interesting for potential drug development.

The absorption of drugs through the oral route can be affected by their susceptibility to efflux mediated by intestinal P-glycoprotein (P-gp). Overexpression of this protein is often a mechanism of drug resistance in *Leishmania*. Thus, the possible inhibitory effect of 2*-n-*propylquinoline on P-gp activity was investigated, at the level of the intestine [82]. Rat everted gut sacs and human intestinal Caco-2 cell lines were used in this study. It was observed that 2*-n-*propylquinoline inhibited P-gp activity with two substrates (rhodamine 123 and digoxin), and two inhibitors (cyclosporin A and verapamil) [82]. These results suggest that 2*-n-*propylquinoline could be associated with another antileishmanial drug in oral treatment to obtain better bioavailability of the second drug by inhibiting P-gp. Furthermore, this also suggests, by analogy, that 2*-n-*propylquinoline could inhibit *Leishmania* ABC transporters, which could help to explain the absence of drug resistance. Although these results remain to be confirmed on *Leishmania*, they suggest that it could be possible to use 2-substituted quinolines to control multi-drug resistance in *Leishmania*. Another advantage is that no cross-resistance was observed between 2*-n-*propylquinoline and amphotericin B, miltefosine or antimonials. When these drug combinations were studied in vitro, the interactions between 2*-n-*propylquinoline and amphotericin B, miltefosine and antimonials were found to be additive [73]. An unpublished metabolomics analysis performed on 2-(2-hydroxyprop-2-enyl)quinoline (Pomel, data not shown) did not identify a specific target, suggesting that the drug could affect multiple targets, which is another factor that could explain the absence of significant drug resistance.

### 3.8. Orientating the Mechanism of Action of 2-Substituted Quinolines: Mechanistic Targeting for a New Series of Compounds

#### 3.8.1. Targeting an Enzyme Involved in Host Cell Recognition

Another strategic approach to valorize 2-substituted quinolines would be to re-direct the series towards a specific target for which the preliminary results are encouraging, since the generation of new inhibitors directed against a leishmania-specific target is an attractive strategy to expand the chemotherapeutic arsenal. GDP-Mannose Pyrophosphorylase (GDP-MP) is an enzyme involved in host–parasite recognition considered to be essential for parasite infection [83,84]. GDP-MPs were purified from *L. mexicana* (*Lm*GDP-MP) and *L. donovani* (*Ld*GDP-MP), and their enzymatic properties were compared with the human enzyme (*h*GDP-MP) [60,85,86,87]. From a rationale design strategy including molecular modeling of 100 potential inhibitors, four compounds were identified as having a promising and specific inhibitory effect on parasite GDP-MP associated with antileishmanial activity. One of them, belonging to the 2-substituted quinoline series, exhibited competitive inhibition on *Ld*GDP-MP [88,89]. This compound, 99 (tetraisopropyl (1-(1-(2-(quinolin-2-ylmethoxy)ethyl)-1H-1,2,3-triazol-4-yl)but-3-yne-1,1-5,3 diyl)bis(phosphonate), showed promising in vitro activity against intramacrophagic amastigotes of *L. donovani*, with an IC_50_ value of 0.63 µM (Table 2) [60]. These encouraging results suggest that compound 99 merits further investigation: in particular, by using nanotechnology to concentrate the molecule in the organs harboring the parasites, particularly the liver in the case of visceral leishmaniasis.

#### 3.8.2. Conferring Chelating Properties on 2-Substituted Quinolines

Chelating agents can inhibit parasite growth, presumably by depriving them of iron, an essential nutrient for cell growth and division. Computational methods were used to explore the Fe^3+^-chelating abilities of a set of quinoline–hydrazone hybrids. A direct relationship between biological activity and the Fe^3+^-chelating ability was observed for these compounds, thereby enriching the range of mechanisms of action of 2-substituted quinolines [90]. In addition, the metabolic stability of compounds can be modulated by their coordination to the heme-iron cytochrome P450 [91]. 

Another series of quinoline derivatives were found to interact with hemin, inhibiting its degradation and generating oxidative stress that could not be counteracted by the antioxidant defense system of the parasite [92].

#### 3.8.3. Obtaining New Metallodrugs

A study evaluated the potential interest of combining 2-substituted quinolines with gold to produce gold(I) complexes, given that metal drugs are an important field of research for antileishmanial drug discovery [93]. In vitro activity was observed for some compounds at submicromolar concentrations on *L. infantum* intramacrophagic amastigotes, with a selectivity index for the best compound of around 10 [93].

#### 3.8.4. Mechanism of Action of Dual Compounds

Metronidazole, an antiprotozoal drug with interesting antileishmanial activity, has been combined chemically with a series of 2-substituted quinolines, leading to metronidazole hybrid compounds [94]. These derivatives were tested against *L. donovani* in vitro and in vivo. They exhibited activity in vitro, with IC_50_ values in the range of 4 to 10 µM, and were effective in vivo on the *L. donovani*/BALB/c mouse model, reducing the parasite burden in the liver and spleen by 80%. The best compound, 15i, triggered oxidative stress that provoked a bioenergetic collapse and apoptosis, as revealed by a decrease in ATP production and the mitochondrial membrane potential [94].

## 4. Antiviral Activities of 2-Substituted Quinolines and the Interest of this Series in Co-Infections

Recently, there has been interest shown in the antiviral properties of molecules based on a quinoline scaffold, particularly for coronavirus infection [95]. Persoons et al. (2021) performed a systematic scan of the anti-coronavirus potential of a range of quinoline-based antimalarial drugs and found broad-spectrum in vitro activity for chloroquine, hydroxychloroquine, mefloquine, ferroquine and amodiaquine [96]. 4-Anilinoquinolines and 4-anilinoquinazolines have been screened against dengue virus, and several active molecules with low toxicity have been identified [97,98]. 

Taking this into account, and since co-infection with leishmaniasis and HIV enhances immunosuppression, it is worth evaluating whether 2-substituted quinolines could provide a double pharmacological benefit by combining an antileishmanial and an antiviral effect. Some of these compounds were first evaluated in vitro at 10 µM against HTLV-1-transformed cells and were active under these conditions [99,100]. Some 2-substituted quinolines were able to downregulate the spontaneous in vitro cell proliferation of HTLV-1-transformed cell lines that is an immunological hallmark of viral infection. Among the 22 compounds evaluated, 4 were found to inhibit spontaneous proliferation by more than 80% at 25 µM [101]. 

Although this level of antiviral activity was not sufficient to merit further investigation of this series as antiviral agents, these preliminary results initiated a study of the effect of 2-substituted quinolines against Ebola virus. There is a need for drugs to treat the disease caused by this pathogen (EVD) [102]. An in vitro screening study evaluating the inhibition of Ebola Zaire replication using a transcription-competent virus-like particle (trVLP) was performed with a library of active compounds. Three 2-substituted quinolines showed IC_50_ values in the range of 1 to 5 µM. This study highlights the potential of quinoline compounds, and particularly 2-substituted quinolines, for the treatment of EVD [102].

## 5. Conclusions

The 2-substituted quinoline series emerged from an ethnopharmacological investigation based on the knowledge of traditional practitioners of the Chimane in Bolivia concerning the treatment of leishmaniasis. From the first isolated natural compounds, several hundred compounds were designed and synthesized, leading to an efficient, safe and cheap chemical series that can be easily synthesized with good yields. 2-Substituted quinolines were active in vitro and in vivo in various experimental leishmaniasis models in BALB/C mice or golden hamsters without toxicity that would limit further development. While structure–activity relationships were not easy to establish, some characteristics that enhanced activity and limited metabolization could be identified, leading to an optimized compound selected by DNDi, designated compound 26 g, that is very active in vitro, with an IC_50_ value of 0.2 µM, but not active enough on the *L. donovani* hamster model to be competitive on the market. Some mechanisms of action of several 2-substituted quinolines have been identified as a function of the substituents on the quinoline scaffold. The possibility of a multitarget mechanism of action could explain the low level of drug resistance obtained after in vitro drug pressure and the absence of cross-resistance, which are both essential criteria for the development of new drugs against leishmaniasis. However, a pharmacoproteomic approach is necessary to identify the biochemical targets in each *Leishmania* species. Some data obtained from a *Galipea longiflora* extract suggest that 2-substituted quinolines could have inhibitory effects in the immune system, but these require further investigation and extension to purified 2-substituted quinolines.

Although some antiviral activity has been observed for this series, it is not strong enough to control both the virus and Leishmania during an HIV/leishmaniasis co-infection. 

A 2*-n-*propyl quinoline salt has been developed to improve oral activity as well as two formulations of the natural compound: liposomes to treat visceral leishmaniasis, and a cyclodextrin formulation to treat cutaneous/disseminated leishmaniasis. All three of these will help to valorize the compound. In parallel, some new synthetic derivatives merit further exploration. 

## Figures and Tables

**Table 1:** In vitro and in vivo antileishmanial activity of some of the most promising 2-substituted quinolines.

Compound	Chemical Formula	In Vitro Activity Expressed as IC50 (µM)			Selectivity	In Vivo Significant Activity Monitored				References
		*L. donovani*	*L. infantum*	*L. amazonensis*	Index = CC50/IC50	on the Leishmania sp./BALB/c Mice Model				
						Oral	Sub-Cutaneous	Intralesional	Intraperitoneal	
2*-n-*propylquinoline	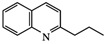	>100 (pro./i.a.)	>100 (i.a.)		/	10 mg/kg/day × 10 (*L. d.*)	85 mg/kg/day × 14 (*L.a*.)	35 mg/kg/day × 15 (*L.a.*)	100 mg/kg/day × 5 (*L. d*.)	Fournet et al., 1993 [42]; Fakhfakh et al., 2003 [55]; Desrivot et al., 2007 [58]; Campos-Vieira et al., 2008 [46]
2*-n-*propylquinoline camphorsulfonic acid	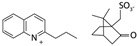	>100 (pro.)	/	/	/	10 mg/kg/day × 10 (*L. d.*)	/	/	/	Campos-Vieira et al. 2011 [59]
2-(2-hydroxyprop-2-enyl)quinoline	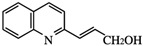	7.8 (pro.)	2	4	> 25	25 mg/kg/day × 15 (*L.a.*); × 10 (*L. i*.); 12.5 mg/kg/day × 5 (*L.d.*)	/	/	/	Campos-Vieira et al., 2008 [46]; Fakhfakh et al., 2003 [55]; Nakayama et al., 2005 [56]
(*E*)-3-quinolin-2-yl-acrylonitrile	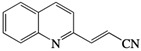	38.6 (pro.); 2.4 (i.a.)	/	/	/	12.5 mg/kg/day × 10 (*L.d*.)	/	/	/	Nakayama et al., 2007 [57]
tetraisopropyl (1-(1-(2-(quinolin-2-ylmethoxy)ethyl)-1H-1,2,3-triazol-4-yl)but-3-yne-1,1-53 diyl)bisphosphonate = Compound **99**	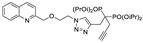	0.63 (i.a.)	/	/	2.4	In progress	/	/	/	Mao et al., 2017 [60]
3-(6- chloro-7-fluoro-4-morpholino) quinoline prop-2-en-1-ol = Compound **26 g**	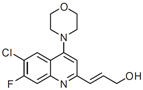	0.22 (i.a.)	/	/	187.5	50 mg/kg/twice daily × 5 (*L.d*.)	/	/	/	Gopinath et al., 2013 [61]
Miltefosine	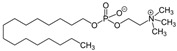	3.6 (pro.); 7.5 (i.a.)	/	/	55	7.5 mg/kg/day × 10 (*L. i.* and *L.d.*)	/	/	/	Campos-Vieira et al., 2008 [46]; Nakayama et al., 2005 [56]; Nakayama et al., 2007 [57]

pro: promastigotes; i.a.: intramacrophage amastigotes.

**Table 2:** In vitro and in vivo antileishmanial activity of liposomal formulations of 2*-n-*propyl quinoline and amphotericin B.

Compound/Formulation	In Vitro Activity on *L. donovani*		Cytotoxicity CC_50_ (µM ± SD) RAW 264.7 Cells	SI = CC_50_/IC_50_	Treatment Regimen (Intravenous Route) × 5 Consecutive Days	Number of Mice	In Vivo Activity Reduction of Parasite Burden (%)
	IC_50_ (µM ± SD) Axenic Amastigotes	Intramacrophage Amastigotes					
2*-n-*PQ-Lip	3.10 ± 0.25 Eq 2*-n-*PQ	5.84 ± 0.31 Eq 2*-n-*PQ	74.09 ± 6.47 Eq 2*-n-*PQ	12.7	3 mg/kg Eq 2*-n-*PQ	8	83.8 ^a^
					1.5 mg/kg Eq 2*-n-*PQ	8	32.5 ^a^
					0.75 mg/kg Eq 2*-n-*PQ	8	5.2
2*-n-*PQ-AmB-Lip	2.02 ± 0.23 Eq 2*-n-*PQ	4.50 ± 0.23 Eq 2*-n-*PQ	58.31 ± 7.32 Eq 2*-n-*PQ	4.3	(1.5 mg Eq 2*-n-*PQ + 0.012 mg Eq AmB)/kg	8	89.0 ^a^
	0.003 Eq AmB	0.006 Eq AmB	0.08 Eq AmB		(0.75 mg Eq 2*-n-*PQ + 0.006 mg Eq AmB)/kg	8	86.5 ^a^
					(0.37 mg Eq 2*-n-*PQ + 0.003 mg Eq AmB)/kg	8	10.3
AmBisome^®^	2.54 ± 0.70 Eq AmB	1.51 ± 0.22 Eq AmB	38.50 ± 2.37 Eq 2*-n-*PQ	25.5	1 mg Eq AmB/kg	8	88.7 ^a^
					0.25 mg Eq AmB/kg	8	27.1
					0.006 mg Eq AmB/kg	8	2.3
Blank liposomes	Inactive	Inactive	/	/	Same suspension	10	5.7
2*-n-*propylquinoline (2PQ)	>100	>100	/	/	/	/	/
Control (vehicle)	Inactive	Inactive	Inactive	/	0.2 mL	12	0

2*-n-*PQ: 2*-n-*propyl quinoline; AmB: Amphotericin B; AmBisome^®^: Liposomal formulation of amphotericin B; 2*-n-*PQ-Lip: Liposomal formulation of 2*-n-*propyl quinoline; 2*-n-*PQ-AmB-Lip: Liposomal formulation of 2*-n-*propyl quinoline and amphotericin B; Eq 2*-n-*PQ: Equivalent 2*-n-*PQ; Eq AmB: Equivalent AmB; SI = Selectivity Index = CC50/IC50 on intramacrophage amastigotes; ^a^ Significant versus control mice: *p* < 0.05.

## Data Availability

Not applicable.
